# Hospitalization costs of patients with severe acute respiratory infections due to COVID-19 in a public teaching hospital: a micro-costing approach

**DOI:** 10.1016/j.bjid.2026.105819

**Published:** 2026-04-27

**Authors:** Ana Carolina Esteves da Silva Pereira, Luciana G. Gallo, Ana Flávia de M. Oliveira, Maria Regina F. de Oliveira, Emanuelly Martins da Silva, Henry M. Peixoto

**Affiliations:** a Universidade de Brasília, Núcleo de Medicina Tropical, Programa de Pós-Graduação em Medicina Tropical, Brasília, DF, Brasil; b Universidade de Brasília, Núcleo de Medicina Tropical, Zika, Arbovirus and other Infections Cohort Studies ‒ ZARICS, Brasília, DF, Brasil; c Fundação Oswaldo Cruz, Gerência Regional de Brasília, Brasília, DF, Brazil; d Instituto Federal de Educação, Ciência e Tecnologia do Tocantins (IFTO), Palmas, TO, Brazil; e Institute for Health Assessment and Translation for Chronic and Neglected Diseases of High Relevance – IATS-CARE, Belo Horizonte, MG, Brazil; f Universidade de Brasília, Ceilândia Sul Campus Universitário, Centro Metropolitano, Brasília, DF, Brazil

**Keywords:** COVID-19, Economic analysis, Hospital costs

## Abstract

**Objective:**

To estimate the costs associated with the hospitalization of patients with Severe Acute Respiratory Infection (SARI) due to SARS-CoV-2 in a university hospital affiliated with the Brazilian Unified Health System (SUS) in 2020 and 2021.

**Methods:**

A bottom-up micro-costing approach was used to conduct a cost analysis based on data from a hospital clinical cohort. Patients with confirmed diagnoses of COVID-19 and SARI, admitted to general wards or intensive care units (with or without mechanical ventilation), were included. Healthcare activities were categorized as hospitalization, ventilation, hemodialysis, nutrition, medications, and laboratory tests. Costs were calculated based on individualized consumption of supplies and procedures, also considering the annualized cost of equipment. Comparisons were made between micro-costing and macro-costing (based on the Hospital Admission Authorizations – AIH).

**Results:**

A total of 627 patients were analyzed. The total cost was R$ 5,824,366.73 (Int$ 2,307,593.80) when healthcare professionals were excluded and R$19,437,731.25 (Int$ 7,701,161.35) when they were included. Medications accounted for the largest share (52.79%). Micro-costing was 1.7-times higher than macro-costing for hospital costs alone and 2.3-times higher when professional costs were included. Multivariate analysis identified length of hospital stay as the main factor associated with increased costs, while age, sex, and referral origin showed no significant association.

**Conclusion:**

This study reveals the substantial economic impact of COVID-19-related hospitalizations in high-complexity care settings. It also highlights the importance of improving financing models for the SUS, particularly given the discrepancies between actual costs and federal reimbursement values. These findings contribute to the development of public policies and response strategies for future health emergencies.

## Introduction

The COVID-19 pandemic presented health systems worldwide with unprecedented challenges, necessitating rapid responses, reorganization of hospital care, and intensive use of resources to address the health crisis.^1^^,^^2^ In Brazil, the Unified Health System (SUS) played a central role in providing care to the population, absorbing the majority of cases, particularly those requiring prolonged hospitalizations in wards and Intensive Care Units (ICUs).^3^^,^^4^ In this context, tertiary teaching hospitals affiliated with the SUS emerged as strategic referral centers for patients with Severe Acute Respiratory Infection (SARI) caused by SARS-CoV-2, playing an essential role in managing the most severe cases.^4^

In the Federal District (DF), the pandemic overwhelmed the public health network, which expanded its care capacity to accommodate the local population and patients from other regions. The University Hospital of Brasília (UHB) was designated as one of the reference centers for managing severe cases, implementing several adaptations such as bed expansion, the creation of COVID-19-specific wards, and the reorganization of care flows. Although these changes were essential to ensuring care during the most critical moments of the pandemic, they also led to a significant increase in hospital costs. This increase reflects the greater complexity of care, the intensive use of life-support technologies, and the high demand for supplies and medications.^5^^,^^6^

The health emergency highlighted the importance of detailed and accurate hospital cost analyses as an essential tool for the efficient allocation of resources.^7^ In the case of hospitalization for severe cases of COVID-19, costs involve multiple components, including expenses related to invasive procedures, ventilatory support, hemodialysis, drug therapies, laboratory tests, and specialized nutrition, whose use varies according to the clinical progression and severity of cases.^8^ These variables make hospital costing in pandemic contexts particularly challenging, reinforcing the need for specific studies to support resource planning and management during crises.^9^^,^^10^ Moreover, the limitation of federal government reimbursements for hospital care, based on fixed values determined by Hospital Admission Authorizations (HAAs), may not reflect the actual costs incurred in patient care, and the COVID-19 pandemic made this limitation even more evident.

Despite the variety of methodologies for hospital cost analysis described in the literature, bottom-up micro-costing is distinguished by its capacity to capture the heterogeneity of care provided by identifying and detailing the components that compose total hospitalization costs.^11^, ^12^, ^13^ By linking clinical information to the actual use of resources, this approach not only allows for a more accurate estimation of costs but also enables the identification of the main drivers of increased hospital expenses during the pandemic. Therefore, it is a valuable tool for guiding preparedness and response strategies for future health emergencies, as well as for supporting public health financing policies.^14^^,^^15^ However, micro-costing depends on institutional context and local cost structures, limiting the generalizability of its findings across different settings.

In this context, this study estimated the costs associated with the hospitalization of SARI patients due to COVID-19 in a teaching hospital affiliated with the SUS during 2020 and 2021.

## Methods

### Study desing

This is a cost analysis based on bottom-up micro-costing. The analysis was developed using a hospital clinical cohort previously established to evaluate the clinical and economic outcomes of SARI caused by COVID-19 at the UHB, as described by Pereira et al.^16^ Micro-costing allowed for the measurement of hospital activity costs based on the frequency of resource use for each patient, in an individualized manner, particularly regarding the use of medical devices, supplies, procedures, food, medications, and laboratory tests during the hospitalization period.

### Setting and study population

This study was conducted at UHB, a public, tertiary-level university hospital located in the Federal District of Brazil and affiliated with the SUS. The hospital was a reference center for COVID-19 hospitalizations during the health emergency and was part of the regulated network of the Federal District Health Department. The complete characterization of the patients, the hospital, and its organization as a reference center for COVID-19 admissions during the health emergency was described by Pereira et al. (2025).

Patients admitted with a confirmed diagnosis of SARI due to COVID-19 were included, based on positive RT-PCR (reverse transcription polymerase chain reaction) and/or IgM serology results recorded in the electronic medical records. Eligibility and the definition of SARI followed clinical criteria validated by physicians on the research team, based on the analysis of hospital records.

UHB adapted its capacity over the pandemic period. Five phases were identified, reflecting changes in care capacity and hospitalization flow: Phase 1 (March to September 2020), Phase 2 (October to December 2020), Phase 3 (January to February 2021), Phase 4 (March to June 2021), and Phase 5 (July to December 2021). According to clinical severity, patients were admitted to one of three hospitalization wards ‒ Yellow Ward (general ward), Orange Ward (ICU without mechanical ventilation), or Red Ward (ICU with mechanical ventilation).

A detailed description of the setting, care structure, and the study population ‒ composed predominantly of male patients, with a median age of 61-years, and a higher proportion of admissions in the Yellow Ward and, in specific phases, in the Orange Ward ‒ as well as the cohort selection criteria, are presented in detail in Pereira et al. (2024). Patient flow dynamics between different hospital wards during phases of the health emergency can be found in Pereira et al.^16^

### Data collection and analysis

The costing process was conducted based on nine steps, inspired by the framework proposed by Etges et al^17^, as shown in Table 1.

**Table 1:** Methodological approaches and steps for conducting the costing process.

Costing Steps	What Was Done
A1. Define the research question	The research question was formulated as follows: What are the costs involved in the hospital care of patients hospitalized with SARI due to COVID-19 at UHB?
A2. Map the care pathway, cost categories, and their respective activities	The clinical care flow was mapped from admission to discharge or death. Cost categories and activities were identified based on this pathway.
A3. Identify the main resources used in each activity	Standard Operating Procedures (SOPs) and information recorded in medical charts were used to list the resources used in each activity, along with their respective quantities.
A4. Identify the unit cost of each resource	The unit costs of the resources used in the activities were obtained from hospital procurement records.
A5. Quantify the execution and frequency of activities	The use and frequency of activities were estimated based on SOPs, interviews with healthcare professionals, medical records, and other institutional systems.
A6. Calculate the total cost per activity	For each activity, the costs of the resources used were multiplied by their quantities, and the results were summed to obtain the total cost of the activity.
A7. Calculate the cost per activity for each patient	The unit cost of each activity was multiplied by the specific frequency of occurrence for each patient, as recorded in the electronic medical records, generating an individualized cost.
A8. Calculate the costs per category of care for each patient	For each patient, the costs of the activities corresponding to each category were summed.
A9. Calculate the total cost per patient and the overall total cost	The total cost per patient was obtained by summing the costs of all categories. The overall total cost of the study was calculated by summing the total costs of all analyzed patients.

The patient flow during hospitalization was mapped (Table 1, Step A2), outlining the possible patient trajectories.^16^ After receiving a confirmed diagnosis of COVID-19, patients could be admitted to the Yellow Ward, the Orange Ward, or the Red Ward according to their clinical severity. As their condition evolved, patients could be transferred between wards, discharged, or referred to other healthcare services.

The study phases were defined based on the analysis of contingency plans developed by the hospital, taking into account the opening and closing of beds dedicated to COVID-19 care according to each emergency response phase (Table 1, Step A2).

From the organization of care activities, six cost categories were defined: hospitalization, ventilation, hemodialysis, nutrition, medications, and laboratory tests (Table 1, Step A2). For the hospitalization, ventilation, hemodialysis, and nutrition categories, the Standard Operating Procedures (SOPs) of the hospital were used to identify the resources, their required quantities per activity, and replacement frequency in days (Table 1, Step A3; Appendix 1). For the medication and laboratory test categories, no additional inputs (e.g., syringes and needles) or materials/equipment used for sample collection and processing were considered, restricting the analysis to the costs of medications and laboratory tests.

The unit costs of the resources listed in Appendix 1 were obtained from procurement reports of purchases made by the hospital between 2020 and 2021. For purchases not identified in these reports, procurement data from the Federal Government Transparency Portal were used^18^ to retrieve purchases made by the hospital in the same period. For the unit costs of laboratory tests, the values available in the SIGTAP Table were used (Table 1, Step A4).

The frequency of activities was obtained from SOPs and interviews with physicians, nurses, nutritionists, and physiotherapists (Table 1, Step A5). Subsequently, the costs of the resources were multiplied by the quantities used in each activity, resulting in the total cost per activity (Table 1, Step A6; Appendix 2).

A data collection form was developed to record activity frequencies. To identify activities performed for each patient, data were extracted from the medical records stored in the Hospital Management Application for University Hospitals (AGHU), version 10.65.42. For medications, prescription records from AGHU containing date-specific medication orders were used. Extraction and processing were performed automatically through a Python script, with support from the ChatGPT tool (version GPT-4.1). The script was developed iteratively, with multiple tests, validations, and manual adjustments, to ensure full compliance with the study protocol criteria (Table 1, Step A6).

The analysis only considered medication prescriptions. Entries containing instructions, clinical notes, supplies, hygiene solutions, or other non-drug items were excluded. Suspended prescriptions were disregarded, and the medication name, formulation, route of administration, and dosage were extracted. Dosage was converted into daily doses. Prescriptions classified as “PRN” or “at physician’s discretion” were considered occasional, while the others were considered regular (Table 1, Step A5).

For the collection of laboratory tests and their quantities per patient, the Complab Advanced Web system (laboratory management system) was used. To handle missing values in laboratory cost variables, multiple imputation was performed using the Predictive Mean Matching (PMM) algorithm via the mice function in R. Imputation was restricted to patients who were actually admitted to the respective ward, as verified by hospitalization time variables. For each ward, specific predictor variables were considered: sex, age, length of stay in the respective ward, and costs per category (hospitalization, ventilation, hemodialysis, nutrition, medication, and professional staff).

The costs of professional care were previously calculated, detailed, and published by the research team in Pereira et al. (2025).^19^ These estimates were derived from an analysis conducted in the same hospital setting and based on the same cohort of patients included in the present study. For each patient, sex, age at admission, clinical outcome, referring unit, symptom duration, length of stay, time spent in each ward, and the quantity or duration of each activity were identified (Table 1, Step A5). The professional costs were estimated using information on staff allocation and workload in the COVID-19 wards and were incorporated into the present analysis to represent the cost of human resources.

The annualization of the artificial ventilator and hemodialysis machine costs considered the acquisition value and an estimated useful life of seven years for both devices. The annual cost was calculated by dividing the acquisition value by the corresponding annualization factor. To estimate the daily cost of use, the annualized value was divided by 365-days, assuming the equipment was available for use throughout the year.^20^^,^^21^

In this study, only artificial ventilators and hemodialysis machines were considered in the estimation of equipment costs. During the study period, these devices were allocated exclusively to COVID-19 wards and were dedicated to the care of patients hospitalized with severe COVID-19. The estimated daily cost was subsequently multiplied by the number of days each patient used the equipment, as obtained from medical records.

Activity costs were calculated by multiplying the resources used by their quantities. These costs were then adjusted for each patient according to the specific frequency of the activities performed. The resulting values were grouped by category of care and summed to estimate the total cost per patient and for the overall sample (Table 1, Steps A7, A8, and A9).

Cost data were initially obtained in Brazilian reais (R$), and they were subsequently adjusted for inflation up to December 2024 using the Broad National Consumer Price Index (IPCA), which represented a 31% cumulative inflation rate over the study period. The adjusted values were then converted into international dollars (Int$ 2024) according to the purchasing power parity (PPP = 2.524) published by the World Bank.

Data analysis and statistical evaluations were conducted using R software (version 4.4.1). Due to the asymmetric distribution commonly observed in healthcare cost data, descriptive statistics were summarized using the median and Interquartile Range (IQR). These estimates were stratified by category, type of activity, ward, and care phase. Data distribution was assessed using the Shapiro-Wilk normality test. For each patient, the total individual cost, as well as the costs associated with each phase and ward, were determined. Furthermore, a comparative analysis was performed between the estimates derived from this study and the costs based on a macro-costing approach, using the proportion of Hospital Admission Authorizations (AIH) attributed to the patients in the analyzed cohort.^16^ AIH values represent administrative reimbursement amounts defined by the Brazilian Unified Health System (SUS) and were used in this study solely for comparison with the costs estimated through the micro-costing approach.

A multivariable regression model was developed to identify the factors associated with total cost. The total cost, which includes professional costs, was considered because they represent one of the main cost components. Since the dependent variable (total cost) exhibited an asymmetric distribution, a Generalized Linear Model (GLM) with a gamma distribution and logarithmic link function was used.

The following independent variables were considered: age, sex, source of admission, reason for discharge, and length of stay in each ward. Variable selection for the final model was guided by the p-value (< 0.10), retaining variables with potential associations with the outcome. Associations with p-values <0.05 were considered statistically significant. The model fit was evaluated using the Akaike Information Criterion (AIC), and the correlation matrix among the predictors was inspected to detect multicollinearity.

The model coefficients were presented in their exponentiated form (eβ), allowing direct interpretation as cost ratios. For categorical variables, eβ represents the ratio between the costs of the category of interest and the reference category, and for continuous variables, it expresses the expected percentage variation in total cost for each additional unit of the analyzed variable.^22^, ^23^, ^24^

### Ethical considerations

This study was conducted in accordance with the ethical principles established for research involving human subjects. The protocol was submitted to and approved by the Research Ethics Committee. Written informed consent was obtained from patients who were conscious and clinically able to provide consent during hospitalization. For severely ill or unconscious patients who were unable to provide consent, informed consent was obtained from a family member via telephone, as authorized by the Research Ethics Committee.

All patients and their families were duly informed about the objectives of the research, the procedures involved, and their rights as participants. Patient identification was carried out by professionals from the hospital’s epidemiological surveillance team, who ensured the confidentiality of the information collected. Strict data protection measures were adopted at all stages of the study to guarantee the anonymity and integrity of the participants’ information.

## Results

A total of 645 individuals hospitalized for SARI due to COVID-19 were considered eligible to participate in the cohort. Of these, 627 patients were included, and data were collected from their electronic medical records. Data from eighteen patients were not collected due to the absence of formal consent to participate in the study.

Most patients were male (59.17%), with a median age of 61-years (IQR 51–73), among whom the 50–69 age group accounted for 45.55% of men and 41.80% of women. The main source of admissions was referrals from other hospitals (76.71%). The number of hospitalized patients varied according to the emergency phase and ward, with 56.6% (n = 355) of patients staying in the Yellow Ward during their hospitalization. Considering the phases separately, Phase 4 had the highest number of admissions to the Orange Ward (n = 157, 56.07%). Regarding clinical outcomes, 28.71% of patients died during hospitalization, and only one case of discharge against medical advice was recorded. A detailed characterization of the analyzed population was previously described in Pereira et al^19^, which addressed the clinical and demographic profile and estimated professional care costs for the same cohort of patients.

The total cost of hospitalizations, including professional care costs, was Int$ 7,701,161.35 (R$ 19,437,731.25), as reported by the research group in Pereira et al. (2025). The median cost was Int$ 6,906.56 (R$ 17,425.96), with an Interquartile Range (IQR) of Int$ 3,203.32–Int$ 14,879.54 (R$ 8,082.28–R$ 37,551.05). The Yellow Ward accounted for the largest portion of costs, Int$ 2,825,131.31 (R$ 7,130,631.42; 36.68%), followed by the Red Ward with Int$ 2,679,474.14 (R$ 6,762,992.73; 34.79%), and the Orange Ward with Int$ 2,196,555.91 (R$ 5,544,107.11; 28.52%). Excluding professional care, the cost was Int$ 2,307,593.80 (R$ 5,824,366.73), with a median of Int$ 1,778.01 (R$ 4,486.80) and IQR of Int$ 800.04–Int$ 4,022.89 (R$ 2,020.81–R$ 10,146.77). It is worth noting that the detailed estimate of professional care costs has already been presented in a previous publication^19^; therefore, the results below, except for the multivariable analysis, focus on the other cost categories.

Among the cost categories analyzed, medications represented the largest proportion, totaling Int$ 1,218,093.76 (R$ 3,074,468.66), which corresponds to 52.79% of the total cost. Laboratory tests accounted for Int$ 252,811.84 (R$ 638,097.08; 10.96%), and food costs totaled Int$ 279,973.47 (R$ 706,653.04; 12.13%). The hospitalization category, which includes activities such as venous access, wound dressings, and catheterizations, totaled Int$ 358,923.02 (R$ 905,921.70; 15.55%). Ventilation procedures represented a cost of Int$ 112,282.41 (R$ 283,400.80; 4.87%), while hemodialysis contributed Int$ 85,509.30 (R$ 215,825.46; 3.71%) (Table 2).

**Table 2:** Costs by category and activity per hospital ward for patients hospitalized with severe acute respiratory syndrome due to COVID-19, University Hospital of Brasília, Brazil, May 2020 to January 2022^a^.

Category e Activity	Yellow Ward		Orange Ward		Red Ward		Total		
	Number of patients	Cost	Number of patients	Cost	Number of patients	Cost	Number of patients	Cost	% of Total
Hospitalization									
Peripheral Venous Access	263	Int$ 7,429.73	212	Int$ 5,035.24	103	Int$ 2,207.69	578	Int$ 14,672.66	0.191
Physical Restraint in Bed	12	Int$ 249.55	7	Int$ 163.50	7	Int$ 60.24	26	Int$ 473.29	0.006
Fluid Balance Monitoring	136	Int$ 11,055.64	110	Int$ 4,668.61	144	Int$ 8,645.30	390	Int$ 24,369.56	0.316
Insulin Pump	5	Int$ 336.52	3	Int$ 197.95	25	Int$ 1,306.49	33	Int$ 1,840.97	0.024
Glycerin Enema	6	Int$ 84.52	6	Int$ 154.95	10	Int$ 84.52	22	Int$ 323.98	0.004
Central Venous Access	70	Int$ 14,372.35	79	Int$ 14,504.20	168	Int$ 51,687.70	317	Int$ 80,564.25	1.046
Wound Dressing	51	Int$ 10,416.43	46	Int$ 6,254.96	54	Int$ 12,714.17	151	Int$ 29,385.57	0.382
Intermittent Urinary Catheterization	6	Int$ 452.85	3	Int$ 61.75	-	-	9	Int$ 514.61	0.007
Indwelling Urinary Catheter	114	Int$ 3,327.56	104	Int$ 2,808.37	175	Int$ 5,616.73	393	Int$ 11,752.66	0.153
Nasoenteric Tube	76	Int$ 1,899.19	78	Int$ 1,922.93	157	Int$ 4,130.74	311	Int$ 7,952.85	0.103
Nasogastric Tube	7	Int$ 565.42	3	Int$ 242.32	6	Int$ 565.42	16	Int$ 1,373.17	0.018
Invasive Blood Pressure Monitoring	29	Int$ 23,212.43	44	Int$ 35,218.86	140	Int$ 127,268.16	213	Int$ 185,699.45	2.411
Total Hospitalization	775	Int$ 73,402.20	695	Int$ 71,233.65	989	Int$ 214,287.16	2459	Int$ 358,923.02	4.661
Ventilation									
Oxygen Catheter	232	Int$ 8,384.59	162	Int$ 5,181.78	86	Int$ 2,812.22	480	Int$ 16,378.60	0.213
Non-Invasive Mechanical Ventilation	76	Int$ 7,412.07	53	Int$ 5,250.21	180	Int$ 29,571.06	309	Int$ 42,233.34	0.548
Invasive Mechanical Ventilation	36	Int$ 11,465.15	49	Int$ 22,000.70	7	Int$ 3,718.43	92	Int$ 37,184.28	0.483
Tracheostomy	‒	‒	20	Int$ 6,212.22	33	Int$ 10,250.17	53	Int$ 16,462.39	0.214
Spirometry	2	Int$ 23.80	‒	‒	‒	‒	2	Int$ 23.80	0.000
Total Ventilation	346	Int$ 27,285.61	284	Int$ 38,644.92	306	Int$ 46,351.88	936	Int$ 112,282.41	1.458
Hemodialysis	‒								-
Hemodialysis	56	Int$ 11,687.46	64	Int$ 17,471.56	106	Int$ 56,350.27	226	Int$ 85,509.30	1.110
Total Hemodialysis	56	Int$ 11,687.46	64	Int$ 17,471.56	106	Int$ 56,350.27	226	Int$ 85,509.30	1.110
Nutrition	‒								
Regular/Normal Diet	91	Int$ 37,964.18	54	Int$ 16,828.30	27	Int$ 5,800.88	172	Int$ 60,593.36	0.787
Liquid Diet	13	Int$ 991.03	10	Int$ 734.10	3	Int$ 183.52	26	Int$ 1,908.66	0.025
Soft Diet	124	Int$ 57,606.77	90	Int$ 34,977.59	71	Int$ 17,976.99	285	Int$ 110,561.35	1.436
Low-Sodium Diet	107	Int$ 40,089.26	81	Int$ 31,416.65	6	Int$ 1,033.82	194	Int$ 72,539.73	0.942
Zero Diet	43	Int$ 0.00	41	Int$ 0.00	88	Int$ 0.00	172	Int$ 0.00	0.000
Parenteral Nutrition	3	Int$ 86.29	1	Int$ 24.65	1	Int$ 110.94	5	Int$ 221.88	0.003
Enteral Nutrition	78	Int$ 1,243.40	87	Int$ 1,102.46	169	Int$ 3,896.24	334	Int$ 6,242.10	0.081
Diabetic Diet	61	Int$ 21,214.32	21	Int$ 5,035.92	2	Int$ 317.22	84	Int$ 26,567.46	0.345
Laxative Diet	‒	‒	3	Int$ 1,217.20	1	Int$ 121.72	4	Int$ 1,338.92	0.017
Total Nutrition	520	Int$ 159,195.25	388	Int$ 91,336.87	368	Int$ 29,441.34	1,276	Int$ 279,973.47	3.635
Medications	‒								-
Medications	327	Int$ 290,313.79	244	Int$ 308,135.63	190	Int$ 619,644.35	761	Int$ 1,218,093.76	15.817
Total Medications	327	Int$ 290,313.79	244	Int$ 308,135.63	190	Int$ 619,644.35	761	Int$ 1,218,093.76	15.817
Laboratory Tests									
Laboratory Tests	355	Int$ 88,733.27	280	Int$ 69,391.61	229	Int$ 94,686.96	864	Int$ 252,811.84	3.283
Total Laboratory Tests	355	Int$ 88,733.27	280	Int$ 69,391.61	229	Int$ 94,686.96	864	Int$ 252,811.84	3.283
Healthcare Professionals									
Healthcare Professionals	355	Int$ 2,174,513.72	280	Int$ 1,600,341.66	229	Int$ 1,618,712.18	584	Int$ 5,393,567.56	70.036
Healthcare Professionals	355	Int$ 2,174,513.72	280	Int$ 1,600,341.66	229	Int$ 1,618,712.18	584	Int$ 5,393,567.56	70.036
TOTAL	2,379	Int$ 2,825,131.31	1,955	Int$ 2,196,555.91	2,188	Int$ 2,679,474.14	6,522	Int$ 7,701,161.35	100

Yellow Ward: General ward. Orange Ward: Intensive Care Unit, without mechanical ventilation. Red Ward: Intensive Care Unit, with mechanical ventilation.

R$, Brazilian reais, official currency of Brazil; Int$, International dollars, adjusted by purchasing power parity (PPP = 2.524, World Bank, 2024) and corrected for inflation (IPCA) up to December 2024. The corresponding cost values in Brazilian reais (R$) are provided in Appendix 3.

“‒”: no activity were recorded in the respective Ward.

a One patient was hospitalized in 2021, with an outcome on January 27, 2022.

The main cost components identified within the analyzed categories included invasive blood pressure monitoring (Int$ 185,699.4501; R$ 468,705.42), central venous access (Int$ 80,564.25; R$ 203,344.16), and fluid balance monitoring (Int$ 24,369.56; R$ 61,508.76) within the hospitalization category. For ventilation, the highest cost was associated with non-invasive mechanical ventilation (Int$ 42,233.34; R$ 106,596.95), followed by invasive mechanical ventilation (Int$ 37,184.28; R$ 93,853.12). Regarding food, soft diets represented the highest cost (Int$ 110,561.35; R$ 279,056.86), while regular diets totaled Int$ 60,593.36; R$ 152,937.65. Hemodialysis, provided to patients with acute kidney failure, generated a total cost of Int$ 85,509.30 (R$ 215,825.46) throughout the study period (Table 2).

When analyzing costs according to clinical outcomes, it was observed that patients who died accounted for the largest share of costs, totaling Int$ 1,111,602.45 (R$ 2,805,684.57), which corresponds to approximately 48.17% of the total cost. Patients who were discharged after clinical improvement accumulated Int$ 975,682.20 (R$ 2,462,621.87), while those transferred to other healthcare facilities or discharged against medical advice presented lower costs (Int$ 220,309.15; R$ 556,066.31) (Table 3).

**Table 3:** Cost by clinical outcome according to category, sex, and age group of patients hospitalized with Severe Acute Respiratory Syndrome due to COVID-19, University Hospital of Brasília, Brazil, May 2020 to January 2022^a^.

	Discharged	Death	Transfer	Discharge Against Medical Advice	Total	% of total
Category						
Hospitalization	Int$ 104,945.28	Int$ 200,076.23	Int$ 53,893.02	Int$ 8.49	Int$ 358,923.02	15.55
Ventilation	Int$ 32,204.52	Int$ 65,324.18	Int$ 14,753.70	‒	Int$ 112,282.41	4.87
Hemodialysis	Int$ 27,370.13	Int$ 49,910.24	Int$ 8,228.93	‒	Int$ 85,509.30	3.71
Nutrition	Int$ 221,445.14	Int$ 32,189.50	Int$ 26,223.96	Int$ 114.87	Int$ 279,973.47	12.13
Medications	Int$ 452,849.59	Int$ 675,927.99	Int$ 89,301.14	Int$ 15.04	Int$ 1,218,093.76	52.79
Laboratory Tests	Int$ 136,867.53	Int$ 88,174.30	Int$ 27,666.61	Int$ 103.40	Int$ 252,811.84	10.96
Total Category	Int$ 975,682.20	Int$ 1,111,602.45	Int$ 220,067.35	Int$ 241.80	Int$ 2,307,593.80	100
Sex						
Female	Int$ 334,601.58	Int$ 509,379.37	Int$ 66,315.97	‒	Int$ 910,296.92	39.45
Male	Int$ 641,080.62	Int$ 602,223.07	Int$ 153,751.38	Int$ 241.80	Int$ 1,397,296.87	60.55
Total by sex	Int$ 975,682.20	Int$ 1,111,602.45	Int$ 220,067.35	Int$ 241.80	Int$ 2,307,593.80	100
Age Group						
0 to 19	‒	Int$ 4493.48	‒	‒	Int$ 4493.48	0.19
20 to 29	Int$ 24,978.24	Int$ 47,368.71	Int$ 6,622.58	‒	Int$ 78,969.52	3.42
30 to 39	Int$ 74,511.18	Int$ 53,627.95	Int$ 33,533.37	‒	Int$ 161,672.50	7.01
40 to 49	Int$ 203,395.35	Int$ 146,306.74	Int$ 11,718.98	‒	Int$ 361,421.07	15.66
50 to 59	Int$ 280,683.28	Int$ 254,110.85	Int$ 39,957.54	‒	Int$ 574,751.67	24.91
60 to 69	Int$ 172,041.11	Int$ 214,574.74	Int$ 28,774.28	Int$ 241.80	Int$ 415,631.94	18.01
70 to 79	Int$ 138,434.68	Int$ 247,695.67	Int$ 57,402.92	‒	Int$ 443,533.27	19.22
80 or older	Int$ 81,638.37	Int$ 143,424.30	Int$ 42,057.67	‒	Int$ 267,120.34	11.58
Total by Age Group	Int$ 975,682.20	Int$ 1,111,602.45	Int$ 220,067.35	Int$ 241.80	Int$ 2,307,593.80	100

R$, Brazilian reais, official currency of Brazil; Int$, International dollars, adjusted by purchasing power parity (PPP = 2.524, World Bank, 2024) and corrected for inflation (IPCA) up to December 2024. The corresponding cost values in Brazilian reais (R$) are provided in Appendix 4.

a One patient was hospitalized in 2021, with an outcome on January 27, 2022; “‒”: no patients were recorded in the respective category.

Male patients were responsible for Int$ 1,397,296.87 (R$ 3,526,777.31; 61%) of the costs, while female patients accounted for Int$ 910,296.92 (R$ 2,297,589.43). The distribution by age group indicated that individuals between 50 and 79-years accounted for the largest share of total costs, particularly those aged 50- to 59-years, who generated Int$ 574,751.67 (R$ 1,450,673.22; 25%) (Table 3).

The analysis of median costs per patient revealed significant differences across wards and pandemic phases. In the Red Ward, median total costs ranged from Int$ 2,992.69 (R$ 7,553.50) in Phase 2 to Int$ 4,627.48 (R$ 11,678.72) in Phase 5. In the Orange Ward, medians varied between Int$ 1,528.46 (R$ 3,859.81) in Phase 3 and Int$ 3,686.12 (R$ 9,301.00) in Phase 1. In the Yellow Ward, median costs were lower, ranging from Int$ 1,164.8815 (R$ 2,939.06) in Phase 2 to Int$ 2,084.34 (R$ 5,259.14) in Phase 4 (Table 4).

**Table 4:** Median costs by hospital ward and emergency response phase according to cost category, University Hospital of Brasília, Brazil, May 2020 to January 2022^a^.

Ward and phase	Hospitalization Cost [A]	Ventilation Cost [B]	Hemodialysis Cost [C]	Nutrition Cost [D]	Medications Cost [E]	Laboratory Tests Cost[F]	Median Total Cost	1st Quartile	3rd Quartile
Yellow Ward									
Phase 1	Int$ 69.49	Int$ 77.24	Int$ 149.12	Int$ 296.96	Int$ 128.87	Int$ 162.49	Int$ 1,965.70	Int$ 707.36	Int$ 4,126.84
Phase 2	Int$ 56.29	Int$ 26.05	Int$ 59.64	Int$ 291.08	Int$ 221.28	Int$ 210.85	Int$ 1,164.88	Int$ 569.43	Int$ 1,799.13
Phase 3	Int$ 46.52	Int$ 52.08	–	Int$ 574.10	Int$ 229.95	Int$ 236.84	Int$ 1,639.17	Int$ 1,178.45	Int$ 2,295.64
Phase 4	Int$ 92.35	Int$ 52.08	Int$ 178.94	Int$ 304.73	Int$ 183.63	Int$ 195.30	Int$ 2,084.34	Int$ 894.01	Int$ 3,888.16
Phase 5	Int$ 71.99	Int$ 52.08	Int$ 149.12	Int$ 287.25	Int$ 148.74	Int$ 179.78	Int$ 1,387.17	Int$ 660.47	Int$ 2,783.11
Total Yellow Ward	Int$ 74.90	Int$ 52.08	Int$ 119.30	Int$ 317.34	Int$ 225.72	Int$ 200.02	Int$ 3,604.75	Int$ 1,809.97	Int$ 7,434.55
Orange Ward									
Phase 1	Int$ 225.74	Int$ 309.94	Int$ 119.30	Int$ 21.23	Int$ 763.65	Int$ 258.61	Int$ 3,686.12	Int$ 2,512.64	Int$ 6,189.15
Phase 2^b^	‒	‒	‒	‒	‒	‒	‒	‒	‒
Phase 3	Int$ 61.05	Int$ 26.05	Int$ 178.94	Int$ 57.46	Int$ 141.54	Int$ 230.13	Int$ 1,528.46	Int$ 710.26	Int$ 2,557.81
Phase 4	Int$ 57.74	Int$ 26.05	Int$ 119.30	Int$ 229.82	Int$ 112.59	Int$ 193.51	Int$ 1,460.61	Int$ 610.11	Int$ 3,109.36
Phase 5	Int$ 72.35	Int$ 103.75	Int$ 238.51	Int$ 512.53	Int$ 237.99	Int$ 301.63	Int$ 3,416.79	Int$ 1,401.98	Int$ 6,270.52
Total Orange Ward	Int$ 71.00	Int$ 52.08	Int$ 119.30	Int$ 172.32	Int$ 300.39	Int$ 212.05	Int$ 2,657.42	Int$ 1,467.64	Int$ 5,239.63
Red Ward									
Phase 1	Int$ 1,117.35	Int$ 154.47	Int$ 417.59	Int$ 24.14	Int$ 738.39	Int$ 418.94	Int$ 4,496.64	Int$ 3,233.26	Int$ 8,431.88
Phase 2	Int$ 987.72	Int$ 103.04	Int$ 387.68	Int$ 44.42	Int$ 356.02	Int$ 322.47	Int$ 2,992.69	Int$ 2,118.32	Int$ 6,406.13
Phase 3	Int$ 235.66	Int$ 64.68	Int$ 298.25	Int$ 182.01	Int$ 179.57	Int$ 275.05	Int$ 3,509.38	Int$ 2,084.34	Int$ 4,285.05
Phase 4	Int$ 963.66	Int$ 103.04	Int$ 238.52	Int$ 69.30	Int$ 393.27	Int$ 278.13	Int$ 4,237.84	Int$ 2,147.85	Int$ 9,519.55
Phase 5	Int$ 911.93	Int$ 154.47	Int$ 238.52	Int$ 45.13	Int$ 614.31	Int$ 356.38	Int$ 4,627.48	Int$ 2,424.28	Int$ 9,002.33
Total Red Ward	Int$ 991.48	Int$ 154.47	Int$ 357.84	Int$ 44.42	Int$ 1,068.21	Int$ 334.85	Int$ 5,033.88	Int$ 2,363.08	Int$ 11,075.88

Yellow Ward: General ward. Orange Ward: Intensive Care Unit, Without mechanical ventilation. Red Ward: Intensive Care Unit, with mechanical ventilation. Phase 1: March to September 2020. Phase 2: October to December 2020. Phase 3: January to February 2021. Phase 4: March to June 2021. Phase 5: July to December 2021; “-”: no patients were recorded in the respective ward and phase.

R$, Brazilian reais, official currency of Brazil; Int$, International dollars, adjusted by purchasing power parity (PPP = 2.524, World Bank, 2024) and corrected for inflation (IPCA) up to December 2024. The corresponding cost values in Brazilian reais (R$) are provided in Appendix 5.

a One patient was hospitalized in 2021, with an outcome on January 27, 2022.

b No records of hospitalizations in this ward and phase.

**Fig. 1:**
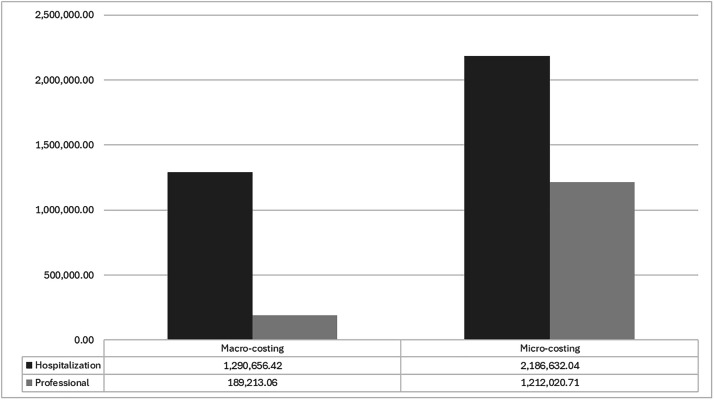
Comparison of hospital and professional costs between macro-costing^a^ and micro-costing approaches for patients hospitalized with Severe Acute Respiratory Syndrome due to COVID-19, University Hospital of Brasília, Brazil, May 2020 to January 2022^b,c^. ^a^ Professional micro-costing results are presented and detailed in Pereira et al.^19^^b^ One patient was hospitalized in 2021, with an outcome on January 27, 2022. ^c^ Results are presented and detailed in Pereira et al^16^,.

A direct comparison between macro-costing^16^ and micro-costing approaches was possible for 95.2% of the samples, covering 597 of the 627 patients included in the study. The macro-costing estimate based on AIH indicated that the total hospital service cost was Int$ 1,290,656.42 (R$ 3,256,516.70), with a median of Int$ 36.34 (R$ 91.70) (IQR Int$ 11.95–94.70; R$ 30.13–R$ 238.94). In contrast, the micro-costing methodology estimated a total cost of Int$ 2,186,632.04 (R$ 5,516,954.65) for the same patients, with a median of Int$ 1,779.38 (R$ 4,490.53) (IQR Int$ 799.61–3,996.20; R$ 2,019.76–R$ 10,086.36), which is 1.7-times higher than the macro-costing value. When incorporating professional costs, the micro-costing estimate was 2.3-times greater than the macro-costing estimate, with total costs of Int$ 3,399,539.45 (R$ 8,578,527.57) and Int$ 1,478,916.31 (R$ 3,732,046.04), respectively (Fig. 1).

In the multivariable analysis, which included professional costs, no statistically significant association was observed between total costs and the variables age, sex, or source of admission. Although female patients showed a tendency toward 32.4% higher costs compared to male patients (eβ = 1.324; 95% CI 0.807–2.197; p = 0.248), the difference was not significant. Similarly, patients transferred to other institutions had 69.6% higher costs compared to those discharged after recovery, but without statistical significance (eβ = 1.696; 95% CI 0.946–3.086; p = 0.094). Age and source of admission did not influence total costs.

On the other hand, length of stay was significantly associated with total hospitalization costs in the Orange and red Wards. Each additional day increased the total cost by 4.9% in the Orange Ward (eβ = 1.049; 95% CI 1.021–1.080; p = 0.003), and 3.4% in the Red Ward (eβ = 1.034; 95% CI 1.012–1.059; p = 0.003). In the Yellow Ward, a similar trend was observed, although the association did not reach statistical significance (eβ = 1.033; 95% CI 1.002–1.066; p = 0.051) (Table 5).

**Table 5:** Multivariable analysis of total costs based on predictors: age, source of admission, discharge reason, hospitalization in the Red Ward, and length of stay in the general ward, Orange Ward, and Red Ward^a^.

Variable	e^β^	95% CI	β	95% CI	p
Age	1.006	(0.992 ‒ 1.019)	0.005	(−0.008 ‒ 0.019)	0.421
Sex					
Male	1	‒	0	‒	‒
Female	1.324	(0.807 – 2.197)	0.280	(−0.214–0.787)	0.248
Origen					
Emergency Care Unit (UPA)	1	‒	0	‒	‒
Other Hospital	1.175	0.690 – 1.950	0.161	(−0.370 ‒ 0.667)	0.545
Other UHB Department	0.899	(0.440 – 1.868)	−0.106	(−0.820 – 0.625)	0.776
Discharge Reason					
Discharged	1	‒	0	‒	‒
Transfer	1.696	(0.946 – 3.086)	0.528	(−0.054 – 1.126)	0.094
Death	1.118	(0.622 – 2.016)	0.112	(−0.474 ‒ 0.702)	0.692
Length of Stay in Red Ward	1.034	(1.012 ‒ 1.059)	0.034	(0.012 ‒ 0.058)	0.003
Length of Stay in Orange Ward	1.049	(1.021 ‒ 1.080)	0.048	(0.022 ‒ 0.077)	0.003
Length of Stay in Yellow Ward	1.033	(1.002 ‒ 1.066)	0.032	(0.002 ‒ 0.064)	0.051

Yellow Ward: General ward. Orange Ward: Intensive Care Unit, without mechanical ventilation. Red Ward: Intensive Care Unit, with mechanical ventilation.

a β, regression coefficient; e^β^, exponential of the regression coefficient (multiplicative effect relative to the reference category); Reference categories are indicated by eβ = 1 and β = 0. Confidence intervals and p-values are not estimated for reference categories and are therefore indicated by “‒”.

## Discussion

The results highlight the complexity of the costs associated with the hospitalization of patients with SARI due to COVID-19 at UHB, emphasizing the influence of factors such as length of stay, discharge reason, and ward of admission. The total cost of hospitalizations was Int$ 2,307,593.80 (R$ 5,824,366.73), excluding professional costs and Int$ 7,701,161.35 (R$ 19,437,731.25) when professional costs were included. Considering costs without professional expenses, the Red Ward ‒ corresponding to the Intensive Care Unit (ICU) with mechanical ventilatory support ‒ accounted for the largest share of costs (45.97%), reflecting the severity of the cases treated. The highest median costs were also observed in this ward. Among the categories analyzed, medications represented the main cost (Int$ 1,218,093.76 [R$ 3,074,468.66]; 52.79% of the total), followed by hospitalization (Int$ 358,923.02 [R$ 905,921.70]; 15.55%) and nutrition (Int$ 279,973.47 [R$ 706,653.04]; 12.13%).

Descriptive analyses indicated that the majority of hospitalized patients were male, which corroborates previous studies that associate greater male vulnerability to progression to severe disease.^25^, ^26^, ^27^ This male predominance (59%) contrasts with the population distribution of the Federal District, where women are the majority, and may be related to biological and behavioral factors, such as differences in immune response and lower adherence among men to preventive measures.^28^ The case fatality rate observed at UHB (28.7%) was similar to the average in the Central-West region (29.0%) and lower than the national rate (32.1%), suggesting that the hospital was able to maintain case fatality rates at levels consistent with its care capacity.^29^^,^^30^

Patients who died during hospitalization accounted for approximately 48% of the total cost, highlighting the significant investment in therapeutic and technological resources, as well as the intensive care efforts made to preserve lives. This finding reflects the high clinical complexity of the cases treated during the pandemic, especially in a context marked by service overload, resource shortages, and high demand for ICU admissions.^31^, ^32^, ^33^, ^34^ The concentration of costs among these patients indicates the level of care required to manage severe cases of SARI due to COVID-19 and underscores the economic impact of hospital care during health emergencies.^35^^,^^36^

In the multivariable analysis, professional costs were considered^16^, and the results showed that age, sex, and discharge reason were not statistically significant factors in determining total hospitalization costs. Although no statistically significant association was observed, slightly higher costs among women were noted. This difference should be interpreted cautiously and may reflect clinical or care-related variables not captured in the present study.^37^^,^^38^

Length of stay emerged as one of the main drivers of increased hospital costs. Significant daily cost increments were observed in the Orange and Red wards, while a similar trend was observed in the Yellow Ward, although the association did not reach statistical significance. This finding is consistent with the literature, which recognizes the duration of hospitalization as a critical factor in the escalation of costs in COVID-19 patients.^35^^,^^38^^,^^39^ Prolonged hospital stays not only intensify resource consumption but also reduce bed turnover, limiting the system’s response capacity in high-demand contexts. Higher costs among patients transferred to other institutions may also reflect clinical requiring referral to facilities with different types of specialization.^4^^,^^33^

Although patients in the Red Ward presented greater clinical severity, higher aggregated nutrition costs were observed in the Yellow Ward. This pattern may be related to the larger number of patients treated in this ward and the more frequent use of oral diets, which presented higher unit costs than enteral or parenteral nutrition (Appendix 2).

The period corresponding to Phase 4, which was associated with the circulation of the Delta variant, was marked by an increase in hospitalizations in the Orange Ward, designated for severe patients without mechanical ventilation. This pattern corroborates the findings in the literature, indicating the strain on hospital systems during epidemic waves.^38^^,^^40^ Despite this, the Red Ward, corresponding to the ICU with mechanical ventilatory support, accounted for 45.97% of the total cost, even though it served a proportionally smaller number of patients, suggesting a correlation between clinical severity and increased hospital costs. Patients admitted in critical condition require greater use of advanced technologies and continuous monitoring, which translates into higher resource consumption per hospitalization day. These findings indicate that the complexity of care is one of the main cost drivers in tertiary hospitals, particularly in high-pressure care settings such as those observed during the pandemic.^41^ These variations demonstrate that the pandemic was not a homogeneous event but a dynamic phenomenon that evolved unevenly over time and across regions, affecting different Brazilian regions in distinct ways and demanding continuous and adaptive care responses.^42^ The pressure on health systems fluctuated according to epidemiological trends, variant circulation, and installed capacity in each phase, which was directly reflected in the organization of services and the composition of hospital costs.^34^^,^^38^^,^^39^ Understanding these dynamics is essential for interpreting the findings of the present study and for guiding future planning and response strategies during health emergencies, particularly regarding budget forecasting, bed allocation, and staffing requirements.^39^^,^^43^^,^^44^

Among the cost categories, medications constituted the largest share, accounting for 52.79% of the total hospitalization costs analyzed. This finding corroborates studies highlight the intensive use of high-cost drugs during the COVID-19 pandemic, including sedatives, neuromuscular blockers, antimicrobials, and anticoagulants used for ventilatory support and the management of infectious complications.^45^^,^^46^ The high proportion of costs attributed to medications may be linked to patient severity but also suggests the need for strategies to optimize the rational use of drugs, both to ensure adequate access and to promote the financial sustainability of the healthcare system.^45^

The comparison between results obtained through micro-costing and macro-costing approaches revealed that micro-costing estimates were substantially higher than those calculated based on AIH. Micro-costing estimates were 1.7-times higher than macro-costing when professional costs were excluded. Furthermore, as demonstrated by Pereira et al. (2025), professional costs alone were 6.4-times higher using micro-costing compared to macro-costing. When hospitalization and human resource costs were combined, micro-costing estimates were 2.3-times greater than those estimated through macro-costing.^19^

It is important to highlight that AIH values represent administrative reimbursement amounts corresponding to federal transfers within the SUS and do not represent the real economic cost of hospital care. Therefore, the comparison performed in this study aims only to illustrate the magnitude of the difference between reimbursement values and the costs estimated through the micro-costing approach. Even with this limitation, these findings reinforce the debate on the underfunding of hospitalizations by the federal government, particularly in the context of high clinical complexity during health emergencies. In the context of the SUS, whose financing structure is tripartite ‒ with responsibilities shared among federal, state, and municipal governments ‒ these discrepancies highlight the necessity to enhance transfer mechanisms and the evaluation of the real costs of hospital care, while respecting the different management levels and their co-responsibility in funding healthcare services.

The values extracted from the AIH reflect only federal transfers and do not cover the entirety of SUS funding, which also includes state and municipal contributions. Despite this limitation, our findings reinforce the discussion on the underfunding of hospitalizations by the federal government and the underestimation of real costs in contexts of high clinical complexity, especially during health emergencies.^47^, ^48^, ^49^ A significant difference was observed between the costs identified and the amounts reimbursed via AIH, indicating the need for supplementation from other funding sources. Although this study was conducted in a federal university hospital in the Federal District, which receives additional transfers from both the federal level and the district government, this reality is not representative of most Brazilian hospital institutions. Consequently, this situation tends to financially burden states and municipalities or, in the absence of sufficient supplementary resources, compromise the quality of care provided to the population.^47^, ^48^, ^49^, ^50^

This study has some limitations that should be considered when interpreting the results. First, the analysis was conducted in a single university hospital linked to SUS, which may limit the generalizability of the findings to other healthcare and regional settings. Although the micro-costing approach allowed for a detailed estimate of the costs associated with hospitalization for SARI due to COVID-19, it has low generalizability and, in this analysis, not all cost components were included ‒ for example, imaging tests, invasive procedures, and administrative costs related to hospital management. The absence of these elements may have led to an underestimation of the real cost per patient. Additionally, the data were extracted from electronic medical records and hospital systems maintained under conditions of high demand and care overload, which may have affected the completeness and accuracy of some records.

The estimation of equipment costs also assumed continuous availability throughout the year to derive a standardized daily cost. Although actual utilization may have varied across different phases of the pandemic, all devices were allocated exclusively to COVID‑19 wards and used to meet the sustained high demand observed in referral hospitals during pandemic scenarios, which supports the plausibility of this assumption. In addition, some resource use frequencies were estimated based on institutional SOPs. Although these protocols reflect the recommended clinical management within the hospital, deviations may have occurred during periods of peak demand and service overload during the COVID-19 pandemic.

Despite these limitations, the results provide robust evidence of the main cost drivers in high-complexity settings and contribute to the discussion on financing and planning health emergency responses in the public health system.

## Conclusion

The present study revealed the high cost associated with the hospitalization of patients with SARI due to COVID-19 in a university hospital of the SUS, highlighting the influence of clinical severity, length of stay, and the complexity of resources used. The predominance of costs related to medications and intensive care underscores the economic impact of hospital care in the context of health emergencies. The discrepancy between the costs estimated through micro-costing and the federal reimbursements via AIH points to the need to improve financing models to more accurately reflect the real costs of hospital care. These findings contribute to the formulation of future crisis responses and provide valuable insights for the ongoing debate on the sustainability of healthcare financing within the SUS.

## Funding

This work was supported by the Ministério da Educação (MEC)/Universidade de Brasília (UnB)/ Faculdade de Medicina (FM) ‒ Integrated research and service actions to face the COVID-19 pandemic in the Federal District ‒ and by grants from the IATS-CARE: Institute for Health Assessment and Translation for Chronic and Neglected Diseases of High Relevance ‒ CNPq/Brazil (408659/2024-6).

## Data availability

The data that support the findings of this study are available from the corresponding author upon reasonable request.

## CRediT authorship contribution statement

**Ana Carolina Esteves da Silva Pereira:** Conceptualization, Methodology, Formal analysis, Data curation, Investigation, Writing – original draft. **Luciana G. Gallo:** Conceptualization, Methodology, Investigation, Writing – original draft. **Ana Flávia de M. Oliveira:** Conceptualization, Methodology, Investigation, Writing – review & editing. **Maria Regina F. de Oliveira:** Conceptualization, Methodology, Writing – review & editing, Project administration. **Emanuelly Martins da Silva:** Formal analysis, Data curation, Investigation. **Henry M. Peixoto:** Conceptualization, Methodology, Formal analysis, Writing – review & editing, Project administration.

## Conflicts of interest

The authors declare no conflicts of interest.

## Appendix Supplementary materials

Image, application 1
