# Supplementary material for: Hospitalization costs of patients with severe acute respiratory infections due to COVID-19 in a public teaching hospital: a micro-costing approach
Source: Braz J Infect Dis. 2026 Apr 27;30(3):105819. doi: 10.1016/j.bjid.2026.105819 (PMC13138186; doi:10.1016/j.bjid.2026.105819)
Supplement: Supplementary file 1 [file mmc1.docx]

BJID-D-25-00323_Supplementary Material

**Appendix 1** Description of the resources considered and length of stay by activity.

| **Categories** | **Activities** | **Resources Considered** | **Replacement Interval (days)** |
| --- | --- | --- | --- |
| Hospitalization | Peripheral Venous Access | PPE, catheter | 3 |
|  | Physical Restraint in Bed | PPE, bandages | N/A |
|  | Fluid Balance Monitoring | PPE, urine collector | N/A |
|  | Insulin Pump | PPE, insulin pump tubing | 3 |
|  | Glycerin Enema | PPE, tube | N/A |
|  | Central Venous Access | PPE, sterile gloves (pair), sterile gauze, disposable syringe, disposable needle, catheter | 7 |
|  | Wound Dressing | PPE, sterile gloves (pair), sterile gauze, film dressing (single use) | N/A |
|  | Intermittent Urinary Catheterization | PPE, sterile gloves (pair), sterile gauze, intermittent catheter | N/A |
|  | Indwelling Urinary Catheter | PPE, sterile gloves (pair), sterile gauze, disposable syringe, indwelling catheter | 15 |
|  | Nasoenteric Tube | PPE, sterile gloves (pair), sterile gauze, 20 mL disposable syringe, nasoenteric tube | 30 |
|  | Nasogastric Tube | PPE, sterile gloves (pair), sterile gauze, 20 mL disposable syringe, nasogastric tube | 14 |
|  | Invasive Blood Pressure Monitoring | PPE, arterial catheter, guidewire, disposable needle, blood pressure transducer, sterile gauze, sterile gloves (pair), 10 mL disposable syringe | 5 |
| Ventilation | Oxygen Catheter | PPE, nasal cannula, oxygen extension tubing (connector tube) | 7 |
|  | Non-Invasive Ventilation | PPE, ventilation mask, ventilation circuit, HEPA filter | 7 |
|  | Mechanical Ventilation (orotracheal or nasotracheal intubation) | PPE, endotracheal tube, mechanical ventilation circuit, filter, tracheal suction cannula, endotracheal tube holder, mechanical ventilator (daily use) | 7 |
|  | Tracheostomy | PPE, tracheostomy cannula with or without cuff, inner tracheostomy cannula, mechanical ventilation circuit, HEPA or HME filter, tracheostomy dressing, tracheostomy collar-type holder | N/A |
|  | Spirometry | PPE, disposable mouthpiece, disposable filter | N/A |
| Hemodialysis | Hemodialysis session | PPE, sterile gauze, procedure gloves, sterile gloves (pair), 20 mL disposable syringe, dialyzer (filter), bloodline set (arterial and venous), arteriovenous fistula needles, film dressing (single use), dialysis machine (daily use) | N/A |
| Nutrition | Regular/Normal Diet | Breakfast, morning snack, lunch, afternoon snack, dinner, and evening snack | N/A |
|  | Liquid |  |  |
|  | Soft |  |  |
|  | Low-sodium |  |  |
|  | Diabetic |  |  |
|  | Laxative diets | Breakfast, morning snack, lunch, afternoon snack, dinner, evening snack, and fruit or laxative cocktail | N/A |
|  | Zero diet | ‒ | N/A |
|  | Enteral nutrition | Formula | N/A |
|  | Parenteral nutrition | Bag and parenteral solution | N/A |
| Medications | Medications (injectable, oral, topical) | Medications administered during hospitalization | N/A |
| Laboratory Tests | Laboratory Tests | Diagnostic and follow-up lab tests performed during hospitalization | N/A |

PPE (Personal Protective Equipment) includes: procedure gloves, surgical mask, gown, and cap.

**Appendix 2** Unit costs by category and activity.

| **Category** | **Activity** | **Unit Costs (R$)** | **Unit Costs (Int$)** |
| --- | --- | --- | --- |
| Hospitalization | Peripheral Venous Access | R$ 16,36 | Int$ 6.49 |
|  | Physical Restraint in Bed | R$ 16,58 | Int$6.57 |
|  | Fluid Balance Monitoring | R$ 14,65 | Int$ 5.81 |
|  | Insulin Pump | R$ 38,14 | Int$ 15.12 |
|  | Glycerin Enema | R$ 13,57 | Int$ 5.38 |
|  | Central Venous Access | R$ 254,05 | Int$ 100.71 |
|  | Wound Dressing | R$ 49,19 | Int$ 19.50 |
|  | Intermittent Urinary Catheterization | R$ 39,66 | Int$ 15.72 |
|  | Indwelling Urinary Catheter | R$ 45,47 | Int$ 18.03 |
|  | Nasoenteric Tube | R$ 45,74 | Int$ 18.13 |
|  | Nasogastric Tube | R$ 155,63 | Int$ 61.69 |
|  | Invasive Blood Pressure Monitoring | R$ 1.542,20 | Int$ 611.34 |
| Ventilação | Oxygen Catheter | R$ 50,17 | Int$ 19.89 |
|  | Non-Invasive Mechanical Ventilation | R$ 148,76 | Int$ 58.97 |
|  | Invasive Mechanical Ventilation | R$ 597,03 | Int$ 236.66 |
|  | Tracheostomy | R$ 598,46 | Int$ 237.23 |
|  | Spirometry | R$ 22,93 | Int$ 9.09 |
| Hemodialysis | Hemodialysis | R$ 114,89 | Int$ 45.54 |
| Nutrition | Regular/Normal Diet | R$ 110,66 | Int$ 43.86 |
|  | Liquid Diet | R$ 70,72 | Int$ 28.03 |
|  | Soft Diet | R$ 110,66 | Int$ 43.86 |
|  | Low-Sodium Diet | R$ 110,66 | Int$ 43.86 |
|  | Zero Diet | R$ 0,00 | Int$ 0.00 |
|  | Parenteral Nutrition | R$ 23,75 | Int$ 9.41 |
|  | Enteral Nutrition | R$ 3,72 | Int$ 1.47 |
|  | Diabetic Diet | R$ 76,40 | Int$ 30.28 |
|  | Laxative Diet | R$ 117,26 | Int$ 46.48 |

**Appendix 3** Costs by category and activity per hospital ward for patients hospitalized with severe acute respiratory syndrome due to COVID-19, University Hospital of Brasília, Brazil, May 2020 to January 2022 (values in Brazilian reais – R$).

| **Category e Activity** | **Yellow Ward** | | **Orange Ward** | | **Red Ward** | | **Total** | |
| --- | --- | --- | --- | --- | --- | --- | --- | --- |
|  | **Number of patients** | **Cost** | **Number of patients** | **Cost** | **Number of patients** | **Cost** | **Number of patients** | **Cost** |
| Hospitalization |  |  |  |  |  |  |  |  |
| Peripheral Venous Access | 263 | R$ 18,752.65 | 212 | R$ 12,708.94 | 103 | R$ 5,572.22 | 578 | R$ 37,033.80 |
| Physical Restraint in Bed | 12 | R$ 629.87 | 7 | R$ 412.68 | 7 | R$ 152.04 | 26 | R$ 1,194.59 |
| Fluid Balance Monitoring | 136 | R$ 27,904.44 | 110 | R$ 11,783.58 | 144 | R$ 21,820.74 | 390 | R$ 61,508.76 |
| Insulin Pump | 5 | R$ 849.38 | 3 | R$ 499.63 | 25 | R$ 3,297.58 | 33 | R$ 4,646.60 |
| Glycerin Enema | 6 | R$ 213.32 | 6 | R$ 391.09 | 10 | R$ 213.32 | 22 | R$ 817.73 |
| Central Venous Access | 70 | R$ 36,275.80 | 79 | R$ 36,608.61 | 168 | R$ 130,459.76 | 317 | R$ 203,344.16 |
| Wound Dressing | 51 | R$ 26,291.07 | 46 | R$ 15,787.53 | 54 | R$ 32,090.57 | 151 | R$ 74,169.17 |
| Intermittent Urinary Catheterization | 6 | R$ 1,143.00 | 3 | R$ 155.86 | - | - | 9 | R$ 1,298.87 |
| Indwelling Urinary Catheter | 114 | R$ 8,398.76 | 104 | R$ 7,088.32 | 175 | R$ 14,176.64 | 393 | R$ 29,663.72 |
| Nasoenteric Tube | 76 | R$ 4,793.55 | 78 | R$ 4,853.47 | 157 | R$ 10,425.98 | 311 | R$ 20,073.00 |
| Nasogastric Tube | 7 | R$ 1,427.13 | 3 | R$ 611.63 | 6 | R$ 1,427.13 | 16 | R$ 3,465.88 |
| Invasive Blood Pressure Monitoring | 29 | R$ 58,588.18 | 44 | R$ 88,892.41 | 140 | R$ 321,224.84 | 213 | R$ 468,705.42 |
| **Total Hospitalization** | **775** | **R$ 185,267.16** | **695** | **R$ 179,793.74** | **989** | **R$ 540,860.80** | **2,459** | **R$ 905,921.70** |
| Ventilation |  |  |  |  |  |  |  |  |
| Oxygen Catheter | 232 | R$ 21,162.71 | 162 | R$ 13,078.82 | 86 | R$ 7,098.05 | 480 | R$ 41,339.58 |
| Non-Invasive Mechanical Ventilation | 76 | R$ 18,708.06 | 53 | R$ 13,251.54 | 180 | R$ 74,637.35 | 309 | R$ 106,596.95 |
| Invasive Mechanical Ventilation | 36 | R$ 28,938.04 | 49 | R$ 55,529.76 | 7 | R$ 9,385.31 | 92 | R$ 93,853.12 |
| Tracheostomy | ‒ | ‒ | 20 | R$ 15,679.65 | 33 | R$ 25,871.43 | 53 | R$ 41,551.08 |
| Spirometry | 2 | R$ 60.08 | ‒ | ‒ | ‒ | ‒ | 2 | R$ 60.08 |
| **Total Ventilation** | **346** | **R$ 68,868.89** | **284** | **R$ 97,539.77** | **306** | R$ 116,992.14 | **936** | R$ 283,400.80 |
| Hemodialysis |  |  |  |  |  |  |  |  |
| Hemodialysis | 56 | R$ 29,499.16 | 64 | R$ 44,098.23 | 106 | R$ 142,228.08 | 226 | R$ 215,825.46 |
| **Total Hemodialysis** | **56** | **R$ 29,499.16** | **64** | **R$ 44,098.23** | **106** | **R$ 142,228.08** | **226** | **R$ 215,825.46** |
| Nutrition |  |  |  |  |  |  |  |  |
| Regular/Normal Diet | 91 | R$ 95,821.60 | 54 | R$ 42,474.63 | 27 | R$ 14,641.42 | 172 | R$ 152,937.65 |
| Liquid Diet | 13 | R$ 2,501.37 | 10 | R$ 1,852.86 | 3 | R$ 463.22 | 26 | R$ 4,817.45 |
| Soft Diet | 124 | R$ 145,399.49 | 90 | R$ 88,283.44 | 71 | R$ 45,373.92 | 285 | R$ 279,056.86 |
| Low-Sodium Diet | 107 | R$ 101,185.29 | 81 | R$ 79,295.64 | 6 | R$ 2,609.36 | 194 | R$ 183,090.29 |
| Zero Diet | 43 | ‒ | 41 | ‒ | 88 | ‒ | 172 | ‒ |
| Parenteral Nutrition | 3 | R$ 217.79 | 1 | R$ 62.23 | 1 | R$ 280.01 | 5 | R$ 560.02 |
| Enteral Nutrition | 78 | R$ 3,138.34 | 87 | R$ 2,782.60 | 169 | R$ 9,834.12 | 334 | R$ 15,755.06 |
| Diabetic Diet | 61 | R$ 53,544.94 | 21 | R$ 12,710.67 | 2 | R$ 800.67 | 84 | R$ 67,056.28 |
| Laxative Diet | ‒ | ‒ | 3 | R$ 3,072.21 | 1 | R$ 307.22 | 4 | R$ 3,379.43 |
| **Total Nutrition** | **520** | **R$ 401,808.82** | **388** | **R$ 230,534.27** | **368** | **R$ 74,309.95** | **1,276** | **R$ 706,653.04** |
| Medications |  |  |  |  |  |  |  |  |
| Medications | 327 | R$ 732,752.00 | 244 | R$ 777,734.32 | 190 | R$ 1,563,982.34 | 761 | R$ 3,074,468.66 |
| **Total Medications** | **327** | **R$ 732,752.00** | **244** | **R$ 777,734.32** | **190** | **R$ 1,563,982.34** | **761** | **R$ 3,074,468.66** |
| Laboratory Tests |  |  |  |  |  |  |  |  |
| Laboratory Tests | 355 | R$ 223,962.77 | 280 | R$ 175,144.43 | 229 | R$ 238,989.88 | 864 | R$ 638,097.08 |
| **Total Laboratory Tests** | **355** | **R$ 223,962.77** | **280** | **R$ 175,144.43** | **229** | **R$ 238,989.88** | **864** | **R$ 638,097.08** |
| Healthcare Professionals |  |  |  |  |  |  |  |  |
| Healthcare Professionals | 355 | R$ 5,488,472.63 | 280 | R$ 4,039,262.34 | 229 | R$ 4,085,629.55 |  | R$ 13,613,364.52 |
| **Healthcare Professionals** | **355** | **R$ 5,488,472.63** | **280** | **R$ 4,039,262.34** | **229** | **R$ 4,085,629.55** |  | **R$ 13,613,364.52** |
| **TOTAL** | **2,379** | **R$ 7,130,631.42** | **1,955** | **R$ 5,544,107.11** | **2,188** | **R$ 6,762,992.73** | **6,522** | **R$ 19,437,731.25** |

Yellow Ward: General ward. Orange Ward: Intensive Care Unit, without mechanical ventilation. Red Ward: Intensive Care Unit, with mechanical ventilation.

R$, Brazilian reais, official currency of Brazil; Int$, International dollars, adjusted by purchasing power parity (PPP = 2.524, World Bank, 2024) and corrected for inflation (IPCA) up to December 2024.

^+^ One patient was hospitalized in 2021, with an outcome on January 27, 2022.

**Appendix 4** Cost by clinical outcome according to category, sex, and age group of patients hospitalized with severe acute respiratory syndrome due to COVID-19, University Hospital of Brasília, Brazil, May 2020 to January 2022^a^ (values in Brazilian reais – R$).

|  | **Discharged** | **Death** | **Transfer** | **Discharge Against Medical Advice** | **Total** |
| --- | --- | --- | --- | --- | --- |
| **Category** |  |  |  |  |  |
| Hospitalization | R$ 264,881.88 | R$ 504,992.41 | R$ 136,025.97 | R$ 21.43 | R$ 905,921.70 |
| Ventilation | R$ 81,284.22 | R$ 164,878.24 | R$ 37,238.35 | ‒ | R$ 283,400.80 |
| Hemodialysis | R$ 69,082.21 | R$ 125,973.44 | R$ 20,769.81 | ‒ | R$ 215,825.46 |
| Nutrition | R$ 558,927.53 | R$ 81,246.30 | R$ 66,189.27 | R$ 289.93 | R$ 706,653.04 |
| Medications | R$ 1,142,992.37 | R$ 1,706,042.25 | R$ 225,396.07 | R$ 37.96 | R$ 3,074,468.66 |
| Laboratory Tests | R$ 345,453.65 | R$ 222,551.93 | R$ 69,830.52 | R$ 260.98 | R$ 638,097.08 |
| **Total Category** | **R$ 2,462,621.87** | **R$ 2,805,684.57** | **R$ 555,450.00** | **R$ 610.30** | **R$ 5,824,366.74** |
| Sex |  |  |  |  |  |
| Female | R$ 844,534.38 | R$ 1,285,673.54 | R$ 167,381.52 | ‒ | R$ 2,297,589.43 |
| Male | R$ 1,618,087.49 | R$ 1,520,011.04 | R$ 388,068.48 | R$ 610.30 | R$ 3,526,777.31 |
| **Total by sex** | **R$ 2,462,621.87** | **R$ 2,805,684.57** | **R$ 555,450.00** | **R$ 610.30** | **R$ 5,824,366.74** |
| Age Group |  |  |  |  |  |
| 0 to 19 | ‒ | R$ 11,341.53 | ‒ | ‒ | R$ 11,341.53 |
| 20 to 29 | R$ 63,045.07 | R$ 119,558.62 | R$ 16,715.39 | ‒ | R$ 199,319.08 |
| 30 to 39 | R$ 188,066.21 | R$ 135,356.94 | R$ 84,638.24 | ‒ | R$ 408,061.40 |
| 40 to 49 | R$ 513,369.86 | R$ 369,278.21 | R$ 29,578.71 | ‒ | R$ 912,226.78 |
| 50 to 59 | R$ 708,444.59 | R$ 641,375.79 | R$ 100,852.83 | ‒ | R$ 1,450,673.22 |
| 60 to 69 | R$ 434,231.75 | R$ 541,586.65 | R$ 72,626.30 | R$ 610.30 | R$ 1,049,055.01 |
| 70 to 79 | R$ 349,409.13 | R$ 625,183.88 | R$ 144,884.97 | ‒ | R$ 1,119,477.98 |
| 80 or older | R$ 206,055.24 | R$ 362,002.94 | R$ 106,153.56 | ‒ | R$ 674,211.74 |
| **Total by Age Group** | **R$ 2,462,621.87** | **R$ 2,805,684.57** | **R$ 555,450.00** | **R$ 610.30** | **R$ 5,824,366.74** |

^a^ One patient was hospitalized in 2021, with an outcome on January 27, 2022.

“‒”, No patients were recorded in the respective category; R$, Brazilian reais, official currency of Brazil; Int$, International dollars, adjusted by purchasing power parity (PPP = 2.524, World Bank, 2024) and corrected for inflation (IPCA) up to December 2024.

**Appendix 5** Median costs by hospital ward and emergency response phase according to cost category, University Hospital of Brasília, Brazil, May 2020 to January 2022^a^ (values in Brazilian reais – R$).

| **Ward and phase** | **Hospitalization Cost [A]** | **Ventilation Cost [B]** | **Hemodialysis Cost [C]** | **Nutrition Cost [D]** | **Medications Cost [E]** | **Laboratory Tests Cost[F]** | **Median Total Cost** | **1^st^ Quartile** | **3^rd^ Quartile** |
| --- | --- | --- | --- | --- | --- | --- | --- | --- | --- |
| Yellow Ward |  |  |  |  |  |  |  |  |  |
| Phase 1 | R$ 175.39 | R$ 194.88 | R$ 376.27 | R$ 749.47 | R$ 325.24 | R$ 410.03 | R$ 4,961.45 | R$ 1,785.61 | R$ 10,410.96 |
| Phase 2 | R$ 142.06 | R$ 65.72 | R$ 150.50 | R$ 734.57 | R$ 558.41 | R$ 532.06 | R$ 2,939.06 | R$ 1,437.09 | R$ 4,541.38 |
| Phase 3 | R$ 117.39 | R$ 131.45 | – | R$ 1,448.65 | R$ 580.29 | R$ 597.54 | R$ 4,136.55 | R$ 2,973.04 | R$ 5,792.37 |
| Phase 4 | R$ 233.11 | R$ 131.45 | R$ 451.52 | R$ 769.06 | R$ 463.36 | R$ 492.77 | R$ 5,259.14 | R$ 2,254.70 | R$ 9,811.37 |
| Phase 5 | R$ 181.69 | R$ 131.45 | R$ 376.27 | R$ 724.82 | R$ 375.28 | R$ 453.59 | R$ 3,500.44 | R$ 1,667.00 | R$ 7,025.10 |
| **Total Yellow Ward** | **R$ 188.95** | **R$ 131.45** | **R$ 301.00** | **R$ 800.67** | **R$ 569.41** | **R$ 504.70** | **R$ 9,093.88** | **R$ 4,565.02** | **R$ 18,756.63** |
| Orange Ward |  |  |  |  |  |  |  |  |  |
| Phase 1 | R$ 569.68 | R$ 782.11 | R$ 301.00 | R$ 53.61 | R$ 1,927.34 | R$ 652.57 | R$ 9,301.00 | R$ 6,339.83 | R$ 15,617.42 |
| Phase 2^b^ | ‒ | ‒ | ‒ | ‒ | ‒ | ‒ | ‒ | ‒ | ‒ |
| Phase 3 | R$ 154.09 | R$ 65.72 | R$ 451.52 | R$ 144.97 | R$ 357.16 | R$ 580.65 | R$ 3,859.81 | R$ 1,792.70 | R$ 6,456.56 |
| Phase 4 | R$ 145.67 | R$ 65.72 | R$ 301.00 | R$ 579.86 | R$ 284.07 | R$ 488.29 | R$ 3,686.52 | R$ 1,540.34 | R$ 7,845.29 |
| Phase 5 | R$ 182.57 | R$ 261.76 | R$ 602.03 | R$ 1,293.67 | R$ 600.70 | R$ 761.30 | R$ 8,621.67 | R$ 3,536.99 | R$ 15,818.60 |
| **Total Orange Ward** | **R$ 179.19** | **R$ 131.45** | **R$ 301.00** | **R$ 434.90** | **R$ 757.88** | **R$ 535.09** | **R$ 6,703.91** | **R$ 3,702.61** | **R$ 13,217.62** |
| Red Ward |  |  |  |  |  |  |  |  |  |
| Phase 1 | R$ 2,818.42 | R$ 389.74 | R$ 1,053.54 | R$ 60.92 | R$ 1,863.16 | R$ 1,056.93 | R$ 11,345.53 | R$ 8,157.35 | R$ 21,279.86 |
| Phase 2 | R$ 2,492.76 | R$ 260.00 | R$ 978.29 | R$ 112.08 | R$ 898.59 | R$ 813.83 | R$ 7,553.50 | R$ 5,345.25 | R$ 16,170.33 |
| Phase 3 | R$ 594.64 | R$ 163.16 | R$ 752.53 | R$ 459.26 | R$ 453.10 | R$ 694.24 | R$ 8,856.61 | R$ 5,259.14 | R$ 10,816.66 |
| Phase 4 | R$ 2,431.97 | R$ 260.00 | R$ 602.03 | R$ 174.82 | R$ 992.34 | R$ 702.02 | R$ 10,689.39 | R$ 5,420.66 | R$ 24,021.66 |
| Phase 5 | R$ 2,300.76 | R$ 389.74 | R$ 602.03 | R$ 113.90 | R$ 1,550.52 | R$ 899.48 | R$ 11,678.72 | R$ 6,118.89 | R$ 22,710.89 |
| **Total Red Ward** | **R$ 2,502.37** | **R$ 389.74** | **R$ 903.04** | **R$ 112.08** | **R$ 2,696.77** | **R$ 844.78** | **R$ 12,699.88** | **R$ 5,961.57** | **R$ 27,941.76** |

Yellow Ward: general ward. Orange Ward: Intensive Care Unit, without mechanical ventilation. Red Ward: Intensive Care Unit, with mechanical ventilation. Phase 1: March to September 2020. Phase 2: October to December 2020. Phase 3: January to February 2021. Phase 4: March to June 2021. Phase 5: July to December 2021.

R$, Brazilian reais, official currency of Brazil; Int$, International dollars, adjusted by purchasing power parity (PPP = 2.524, World Bank, 2024) and corrected for inflation (IPCA) up to December 2024.

^a^ One patient was hospitalized in 2021, with an outcome on January 27, 2022.

^b^ No records of hospitalizations in this ward and phase.
